# Cross-linguistic evidence for gender as a prominence feature

**DOI:** 10.3389/fpsyg.2015.01356

**Published:** 2015-09-08

**Authors:** Yulia Esaulova, Lisa von Stockhausen

**Affiliations:** Department of Psychology, University of Duisburg-Essen, Essen, Germany

**Keywords:** prominence, grammatical gender, stereotypical gender, thematic roles, grammatical functions

## Abstract

This paper discusses recent findings in the online sentence processing research that suggest to consider gender information a prominence feature. Prominence features are hierarchically ordered information types that interact with formal features of arguments (e.g., grammatical functions, thematic roles) and thus determine the readers’ ability to efficiently interpret linguistic ambiguities. While previous research addressed a number of prominence features (e.g., animacy, definiteness, person), there is now first empirical evidence indicating that gender information also influences the assignment of thematic roles across languages. Grammatically masculine role nouns are processed faster as agents than patients compared to feminine ones. Stereotypically male role nouns (e.g., electrician) are integrated with an agent role easier than neutral ones (e.g., musician), which in turn are integrated easier than female ones (e.g., beautician). Conceptualizing gender as a prominence feature will not only expand our knowledge about information types relevant for online comprehension but also uncover subtle gender biases present in language. The present work explores the possibility for a theoretical integration of social psychological and psycholinguistic research focusing on gender with research on prominence. Potential advantages an interdisciplinary approach to the study of gender as a prominence feature, open questions and future directions are discussed.

## Introduction

Natural languages often present their users with ambiguities that require an interpretation even in cases when the provided information does not suffice to resolve them. Comprehenders may apply one of the two major strategies to process the ambiguous linguistic input. One strategy would involve computational mechanisms that defer hypotheses about the possible meaning of a sentence until enough information is provided to resolve ambiguities. Another strategy would involve processing the sentence incrementally, on a word-by-word basis, as the linguistic input unfolds. While both strategies have the same goal, the incremental integration seems to offer a more efficient and rapid way to achieve the interpretation of a sentence, most certainly for languages where the (disambiguating) verb occurs in sentence- or clause-final position (Kamide et al., 2003). The model of incremental processing assumes that language users make probabilistic predictions about the syntactic structure and the meaning of a sentence based on a number of constraints (e.g., case, agreement). Prominence is a theoretical notion that is used to identify certain information types as constraints (or prominence features) that are organized hierarchically and interact with formal features of verbal arguments (de Hoop and Lamers, 2006). As a result of this interaction, in the process of incremental interpretation thematic structure and grammatical functions of verbal arguments can be predicted from the position of their prominence features on a scale, where higher ranked (more prominent) arguments are more likely to be interpreted as agents and subjects rather than patients and objects. Animacy, definiteness, and person are considered such hierarchically organized information types and are referred to as prominence features (Lamers, 2012). Thus, prominence features are discussed in terms of scales, where animate entities are more prominent and outrank inanimate, definite outrank indefinite, and first and second person outrank third. Readers rely on prominence information as a cue to assess the structure and the meaning of a sentence especially in cases where information from case marking and/or word order is ambiguous and cannot be used for interpretation. In this paper, we propose to conceptualize gender information as another prominence feature, i.e., the information type that systematically affects readers’ predictions about thematic roles and grammatical functions of arguments. We both suggest a theoretical foundation and evaluate the existing empirical evidence for different types of gender information to function as a prominence feature with the aim to demonstrate that gender influences go beyond the well-known agreement and mismatch effects.

## Prominence and Sentence Structure

The role of prominence features for the comprehension of sentences is often discussed in terms of their interaction with grammatical functions or thematic roles. Research has shown that the relative ease or difficulty in the assignment or accessibility of entities performing an action (i.e., grammatical subjects or thematic agents) and those receiving an action (i.e., grammatical objects or thematic patients) depends on the characteristics of prominence features they possess. Generally, entities possessing highly ranked prominence features tend to occupy more syntactically prominent positions, while entities with lower rankings in terms of prominence occupy less prominent syntactic positions. In case of animacy as a most widely studied prominence feature, for instance, animate nouns or noun phrases are rather associated with subject functions and agent roles and inanimate ones with object functions and patient roles (e.g., MacDonald, 1994; Traxler et al., 2002; Bornkessel-Schlesewsky and Schlesewsky, 2009). In other words, prominence hierarchies (e.g., animates over inanimates) and the hierarchies within grammatical functions (e.g., subjects over objects) and thematic roles (e.g., agents over patients) align with or map onto each other. This often results in the so-called “harmonic alignment” (Aissen, 2003) when highly prominent entities (e.g., animates) are matched with highly ranked grammatical functions/thematic roles (subjects/agents) and less prominent entities (e.g., inanimates) are matched with lower ranked objects/patients. Arguments which prominence features are harmonically aligned with their thematic roles/grammatical functions have been shown to be processed faster and even facilitate comprehension difficulties related to syntactic ambiguities (e.g.,Traxler et al., 2002, 2005; Gennari and MacDonald, 2008). The theoretical substantiation of the principle of harmonic alignment is offered by the model of Incremental Optimization of Interpretation (de Hoop and Lamers, 2006). This model defines several constraints (e.g., agreement, case, etc.) that are used to distinguish subjects from objects (e.g., the verb agrees with the subject and not the object; the subject is in the nominative case, while the object is in the accusative case, etc.). Prominence is defined as one of these constraints and assumes subjects to be associated with higher prominence rankings than objects. In this respect, prominence can be seen as a semantic cue that links grammatical functions to semantic relations between arguments during language comprehension. It is worth noting that prominence has been shown not only to relate formal structure of a sentence to its semantic content, but also to modulate the interpretation of a sentence. One of the examples of such modulation that is widely discussed in literature concerns the interpretation of sentences with subject- and object-extracted relative clauses. It is a well-established finding that subject-extracted relative clauses (e.g., *The reporter that attacked the senator admitted the error*) are easier for comprehension than object-extracted ones (e.g., *The reporter that the senator attacked admitted the error*; e.g., King and Just, 1991). However, Traxler et al. (2002) demonstrated that the difficulty in the processing of relative clauses can be modulated as a function of animate vs. inanimate sentence heads. When sentence heads were inanimate (*The movie that the director watched received the prize*), object-extracted clauses were almost as easy to comprehend as their subject-extracted counterparts (*The director that watched the movie received the prize*). These and similar findings indicate that animacy as a prominence feature is a semantic cue that may reduce or strengthen syntactic complexity effects and thus is a factor significantly influencing the comprehension of a sentence together with syntactic and thematic structures (Traxler et al., 2005).

## Gender as a Prominence Feature: Theoretical Motivation

The interaction of animacy with thematic roles and grammatical functions has been confirmed as a cross-linguistic phenomenon in a number of linguistic tasks other than the interpretation of relative clauses (e.g., English—McDonald et al., 1993; German—Van Nice and Dietrich, 2003; Spanish—Prat-Sala, 1997) and established animacy as a prominence feature. Describing animacy as a prominence feature, Yamamoto (1991) regards it as a “supra-linguistic” concept—a fundamental semantic dimension which as such also affects a number of linguistic phenomena (e.g., word order, case marking). This understanding of a prominence feature as a supra-linguistic concept can be applied to a number of information types other than animacy and we would like to argue that gender is one of them. Similarly to animacy, gender is a fundamental semantic dimension expressed on a biological level as a characteristic of individuals and on a social level through social practices (Ridgeway, 2001). As one of the categories essential for social interaction (Fiske, 1998), gender is represented in language in diverse ways: through grammatical gender (i.e., a noun class system where gender may be identified by grammatical markings, such as feminine suffixes, as in German *Musiker*_masculine_ vs. *Musikerin*_feminine_ “musician”), natural gender (i.e., referring to the sex of a referent, as in pronouns), definitional gender (i.e., where it is part of the definition of the word, as in *king* vs. *queen*) and stereotypical gender of role nouns (i.e., the likelihood that an activity would be done by either a man or a woman that reflects existing gender stereotypical representations, as in stereotypically male *electrician* vs. stereotypically female *beautician* vs. neutral *musician*; e.g., Gabriel et al., 2008; Kreiner et al., 2008)^1^. The way both biological and social aspects of gender are represented in language makes it plausible to consider gender information—denoted on grammatical (grammatical gender) and conceptual (stereotypical gender) levels in role nouns—a prominence feature influencing the interpretation of thematic roles in a sentence. Whereas there is still no clear understanding which communicative function gender serves, Bates et al. (1996) suggest that it does serve one considering how pervasive and persistent it is in the world’s languages despite its linguistic costs. In their experiment, Bates et al. (1996) demonstrated that grammatical gender of an adjective in Italian clearly primed the recognition of the following noun. Some research has found that language users may not always choose the interpretation strategy that would focus on gender information as useful (e.g., Garnham et al., 1992; McDonald and MacWhinney, 1995), however most research has shown that the integration of gender information represented in language is crucial for comprehension and is processed in highly automatized ways (e.g., Irmen, 2007; Cacciari et al., 2011; Esaulova et al., 2014). This research identified various types of gender information (e.g., grammatical gender markings, gender stereotypical representations, definitional gender), as well as the time course of its integration during language processing. To identify influences of different types of gender information person denotations (e.g., *electrician* or *soccer fan*) are often used, since they both entail grammatical gender information (marked morphologically or by the determiner) and are subjects to gender stereotypes (Baudino, 2001). Research paradigms involving such denotations, or role nouns, typically employ reference resolution, which requires the integration of gender information in order to be interpreted (e.g., a masculine or feminine pronoun *he/she* referring to stereotypically masculine role noun *electrician*). This integration may result in mismatch effects that are observed in cases of grammatical disagreement and other gender incongruities (e.g., *electrician*—*she*) and are reflected in longer processing times indicated by corresponding behavioral measures (e.g., longer fixation times and more regressions in case of eye-tracking measures). Thus, in their reading time study, Kennison and Trofe (2003) presented readers with pairs sentences, where the first one contained a stereotypically male or female role noun and the second one a pronominal reference to this role noun (*he/she*). The results showed significantly longer reading time when the stereotypical gender of the role noun and the pronoun gender mismatched (e.g., *executive*_Male_ … *she*; *secretary*_Female_ … *he*) compared to when they matched (e.g., *executive*_Male_ … *he*; *secretary*_Female_ … *she*).

In a similar vein, Duffy and Keir (2004) monitored participants’ eye-movements when they read sentences like *a babysitter found himself/herself humming while walking up to the door*, where stereotypical gender of a role noun either matched or mismatched the reflexive pronoun referring to it (Experiment 1). The results showed the gender mismatch effect when reflexive pronouns were incongruent with the gender stereotype. Interestingly, this effect disappeared in Experiment 2, where a context preceding sentences disambiguated the gender of a character explicitly stating whether it was a man or a woman. Kreiner et al. (2008) further explored instances when the gender mismatch effect can be overridden. After demonstrating that readers slow down when an anaphor (e.g., *herself*) mismatched stereotypical (e.g., *minister*) and definitional (e.g., *king*) gender of the role noun antecedent (Experiment 1), they contrasted the congruity of stereotypical and definitional gender with the reflexive in cataphora sentences, showing that when the reflexive preceded the role noun, the mismatch effect only occurred for definitional but not stereotypical gender nouns (Experiment 2). Kreiner et al. (2008) interpret the results as supporting theoretical perspectives on the nature of different gender types and argue that stereotypical gender is inferred from world knowledge, as suggested by the mental models approach, while definitional gender is defined lexically.

Another theoretical approach was addressed by Sturt (2003), who demonstrated the gender mismatch effect in an eye-tracking study that used paragraphs containing two potential antecedents—one of them a stereotypically male or female role noun—for the reflexive anaphor (e.g., *Jonathan/Jennifer was pretty worried at the City Hospital. He/She remembered that the surgeon had pricked himself/herself with a used syringe needle. There should be an investigation soon.*). Chomsky’s binding theory (Chomsky, 1981) predicts that the second character (*the surgeon*) is a grammatical (and the only possible) antecedent, while the character mentioned first is an ungrammatical one. Even though an early effect between the grammatical antecedent and the anaphor supported the binding theory, ungrammatical antecedents also affected processing at a relatively later stage.

Some research reporting gender mismatch-effects detected asymmetries in the processing of gender cues. In an eye-tracking experiment (Experiment 2) in German, Reali, Esaulova and von Stockhausen (2015) analyzed the resolution of pronoun anaphors (*er* “he”/sie “she”) referring to gender-stereotypical descriptions of an occupation (e.g., stereotypically male M. F. repariert und stellt Möbel her, arbeitet mit Holz. “M. F. repairs and produces pieces of furniture, works with wood.”). Results revealed an asymmetry in the processing of anaphor gender, as the mismatch effect occurred earlier for masculine and later for feminine pronouns, suggesting that representations of female referents are more flexible and thus are integrated easier into counterstereotypical contexts compared to male referents. In a priming study, Cacciari and Padovani (2007) reported an asymmetry in the same direction on bigender nouns, where the mismatch effect manifested for masculine pronouns following stereotypically female role nouns (e.g., *insegnante*—*lui* “teacher—he”) but not for feminine pronouns after male roles (e.g., *ingegnere*—*lei* “engineer—she”). Using event-related potentials (ERPs), Siyanova-Chanturia et al. (2012) identified an N400-like effect as an electrophysiological response to masculine but not feminine pronouns primed by stereotypically incongruent role nouns (e.g., insegnante—lui “teacher—he”). Taking into account the results of the three aforementioned studies, it must be noted that considering specific gender cues (i.e., masculine vs. feminine) can prove beneficial to the understanding of the effects related to gender agreement or congruity.

As some other studies indicate, gender agreement seems to affect language comprehension in ways that go beyond word recognition and anaphor resolution but can also be used as a cue to determine thematic roles, even though gender differs from other aspects of inflectional morphology (e.g., case, person, number, etc.) in that it is an inherent property of nouns. Friederici and Weissenborn (2007) provide an overview of ERP studies demonstrating that subject-verb gender agreement is among other features (number, person) that elicit left anterior negativity effects in the identification of thematic structures. Devescovi et al. (1998) and Kail (1989) also argue that gender agreement seems to play a role in determining “who did what to whom” in a sentence and that the extent to which it is used as a cue may depend on the age of language users and the language itself. This is in line with the competition model (MacWhinney et al., 1984), which evaluates the extent to which readers rely on different cues—word order, (gender) agreement, animacy, etc.,—to interpret the structure of a sentence and predicts that the strength of each of the cues varies across languages. Importantly, the focus of these works is on gender as one of the cues used to determine thematic roles (along with case and word order) and consider gender agreement (e.g., between a noun and a verb) rather than specific gender characteristics of nouns. The question whether particular gender characteristics could make one (role) noun fit a thematic role better than another noun has so far remained open. Thus, while the influences of gender information on language comprehension have been repeatedly demonstrated using various paradigms, research methods and theoretical approaches, considering gender a prominence feature would predict gender to influence the processing of formal relations in a sentence structure on a much more far reaching, rather implicit level. In line with this idea, previous research on biases (discussed in more detail below) has shown that the use of particular linguistic structures (e.g., negations) implies beliefs and expectations corresponding to existing stereotypes (e.g., de Villiers and Flusberg, 1975; Beukeboom et al., 2010). Understanding whether and how gender information is used to predict thematic structures could provide an insight on mechanisms underlying gender biases and stereotyping.

Further indications for why gender information may need to be considered as a prominence feature come from two, at first sight, theoretically distinct areas of research. On the one hand, linguistic theories, such as differential object marking (Aissen, 2003) suggest that the overt case marking of an object reflects its place on a prominence hierarchy, where overtly case-marked objects are more prominent than non-marked ones. In languages with grammatical gender system, case marking often depends on grammatical gender and differs for feminine and masculine entities. In German, for instance, the singular form of the masculine determiner is marked overtly in all four cases (*der*_Nominative_; *den*_Accusative_; *dem*_Dative_; *des*_Genitive_), while the singular form of the feminine determiner only has two forms: one for nominative and accusative cases and one for dative and genitive (*die*_Nominative/Accusative_; *der*_Dative/Genitive_). According to the differential object marking theory, such differences in case markings indicate a hierarchy where grammatically masculine and feminine entities differ in rankings. The differential marking of grammatical gender information suggests that gender may be considered a prominence feature for which hierarchical organization is typical. On the other hand, research in social cognition relates masculinity and femininity to constructs of status and power, which are described in terms of high and low extremes (high vs. low status/power). Higher rankings on these hierarchies tend to be attributed to masculinity, therefore indicating a gender hierarchy where masculinity outranks femininity (e.g., Spence and Buckner, 2000; Ridgeway, 2001; Koenig et al., 2011). Furthermore, thematic agents reflect to some extent the social psychological agency understood as a modality of human behavior and expressed in the desire to master the environment and experience competence, power, and achievement (Bakan, 1966). This social concept of agency—in turn—relates to gender through sex role characteristics that differ for men and women and become apparent through distinct socialization patterns. The tendency to be socialized to be achievement-oriented, independent, and self-sufficient, for instance, is reported to be typical for men but not women (Cross and Madson, 1997). Similar to the social-role theory, expectation states theory proposes that the gender system is entwined with social hierarchy and leadership through status beliefs (Wagner and Berger, 1997). Status beliefs are commonly held cultural beliefs that associate greater competence and social significance with men than women and are at the core of gender stereotypes (Williams and Best, 1990). Thus, hierarchies within the social gender system that are related to leadership, status, and power can be viewed as representing agency on a social psychological rather than linguistic level of a thematic structure.

The hierarchical organization within the concept of gender suggested by the described linguistic and social psychological phenomena invites an interdisciplinary approach to consider whether gender information as it is represented in languages can be conceptualized as a prominence feature. If this is the case, then grammatical and stereotypical gender of role nouns should affect the processing of their thematic roles in sentences. When the thematic structure of a sentence is ambiguous and allows for more than one interpretation of who produced or received an action, more prominent role nouns (e.g., grammatically masculine and stereotypically male) should be perceived as better agents and less prominent ones (e.g., grammatically feminine and stereotypically female) as better patients. If gender information functions as a prominence feature, differences in the processing of role nouns in specific thematic roles (agent or patient) depending on their gender characteristics should be observed, as some role nouns would be seen as fitting their thematic roles better than others. This hypothesis was addressed in two studies in German and French languages, which identified gender hierarchies that affect the processing of thematic structures. These studies and their experimental results are described below.

## Overview of Studies on Gender as a Prominence Feature

The first study (Esaulova, 2015) includes two eye-tracking experiments (*N*_1_ = 32; *N*_2_ = 40) in German, where locally ambiguous subject- and object-extracted relative clauses were used to examine whether gender information may influence the identification of role nouns as agents and patients in sentences. Readers were presented with sentences like *Die Flugbegleiterin, die viele Tourist-en/-innen beobachtet hat/haben, ist aufmerksam* “The flight attendant_Typically female + feminine_, who has observed many Tourists_Neutral + feminine/masculine_/whom many tourists_Neutral + feminine/masculine_ have observed, is attentive.” These sentences were designed in such a way that agent and patient roles remained ambiguous until readers reached the very final word of the relative clause—the auxiliary verb *hat/haben* “has/have,” which then disambiguated agent and patient roles through its number marking. In Experiment 1, all used role nouns were neutral with regard to stereotypical gender. In terms of grammatical gender, main clause role nouns varied and were either grammatically feminine or masculine, while relative clause role nouns were feminine and did not vary. The experimental design thus included grammatical gender of the main clause role noun (RN1) and the relative clause type as factors, which resulted in four conditions: 1. masculine RN1 + SRC; 2. feminine RN1 + SRC; 3. masculine RN1 + ORC; 4. feminine RN1 + ORC. Depending on the type of the relative clause, RN1 served either as a thematic agent or as a patient. According to the hypothesis, grammatically masculine agents were expected to require shorter processing times compared to feminine ones (conditions 1 vs. 2), while masculine patients were expected to require longer processing times compared to feminine ones (conditions 3 vs. 4). The results showed shorter reading times^2^ when masculine rather than feminine role nouns served as agents (conditions 1 vs. 2), while no differences were found when the two role nouns served as patients (conditions 3 vs. 4). In Experiment 2, main clause role nouns varied in stereotypical gender and were either stereotypically female (e.g., flight attendant) or neutral (e.g., student), while their grammatical gender was feminine. Relative clause role nouns varied in grammatical gender and were either masculine or feminine, while they were neutral with regards to stereotypical gender. The experimental design included stereotypical gender of the main clause role noun (RN1), grammatical gender of the relative clause role noun (RN2) and the relative clause type as factors. This resulted in either stereotypically female or neutral RN1 in one of the four conditions: 1. masculine RN2 + SRC; 2. feminine RN2 + SRC; 3. masculine RN2 + ORC; 4. feminine RN2 + ORC. In addition to the hypothesis regarding grammatical gender of RN2 (identical to that in Experiment 1), hypothesis concerning stereotypical gender predicted longer reading times for stereotypically female than neutral agents and for neutral than female patients. The results of Experiment 2 again showed that reading times were shorter for grammatically masculine rather than feminine role nouns when they served as agents (conditions 3 vs. 4) but no significant differences were detected when they served as patients (conditions 1 vs. 2). On the other hand, the effect of stereotypical gender emerged for both agents and patients, with longer reading times for stereotypically female than neutral agents and for neutral compared to female patients. These findings suggest that both grammatical and stereotypical gender is involved in the processing of thematic relations in a sentence, namely the interpretation of agents and patients. Differences in the processing times indicate the relevance of both types of gender cues for the identification of what thematic role a role noun serves in a sentence.

Another study (Esaulova, 2015) also included two eye-tracking experiments (*N*_1_ = 25, *N*_2_ = 33) that used the French gender-ambiguous indirect object pronoun *lui* “him/her” as a backward anaphor to investigate whether gender information may affect the processing of grammatical functions/thematic roles of role nouns. The pronoun *lui* “him/her” in sentences like *En vérité, la diététicienne lui a recommandé, donc à ce/cette pharmacien/pharmacienne, un plan rigoreux* “In fact, the dietician_Typically Female + feminine_ recommended to him/her_gender – ambiguous_, so to this_masculine/feminine_ pharmacist_Neutral + masculine/feminine_, a strict plan” indicated an upcoming referent without specifying its gender. According to the design, referent role noun varied in grammatical gender (masculine or feminine) and was neutral with regard to stereotypical gender. The first role noun had a fixed grammatical gender (feminine in Experiment 1 and masculine in Experiment 2) and varied in stereotypical gender (female/neutral in Experiment 1 and male/neutral in Experiment 2). Hypotheses predicted longer processing time for grammatically masculine than feminine objects/patients (the second role noun), as well as neutral than stereotypically male and stereotypically female than neutral subjects/agents (the first role noun). The results of both experiments showed longer reading times for grammatically masculine compared to feminine objects/patients, as expected by the hypothesis regarding the grammatical gender of role nouns. They also supported predictions about the stereotypical gender of role nouns showing longer reading times for stereotypically female than neutral (Experiment 1) and neutral than stereotypically male subjects/agents (Experiment 2). The findings demonstrate a relative difficulty in the processing of masculine compared to feminine referents in both experiments, which indicates that readers do create specific expectations about the gender of the referent role noun relying on its grammatical function of an object in the sentence. Additionally, the findings suggest an easier integration of neutral rather than stereotypically female (Experiment 1) and stereotypically male rather than neutral nouns (Experiment 2) with an subject/agent role in a sentence.

## Evidence-Based Interpretation of Gender as a Prominence Feature

The findings of these two studies provide the first evidence that grammatical and stereotypical gender information in role nouns may be conceptualized as a prominence feature. Like other prominence features, gender information appears to map onto thematic relations and grammatical functions in sentences. The principle of harmonic alignment makes it possible to identify whether an information type is organized hierarchically and how its components are ranked on this scale through the relative ease or difficulty in the processing. When the ranking of the feature on one prominence scale differs with the ranking on another prominence or thematic roles/grammatical functions scale, processing costs are higher compared to when scales are aligned with each other, so that the rankings on one correspond to the rankings on the other and are both either high or low. Esaulova (2015) demonstrated a relative ease in the processing of sentences with relative clauses when masculine rather than feminine grammatical gender of role nouns corresponded to high-ranked thematic agents. Similarly, yet in a different language, the processing of sentences with backward anaphors was easier when low-ranked object referents were grammatically feminine rather than masculine (Esaulova, 2015). Both of these findings suggest the hierarchical organization of grammatical gender information, where masculine gender outranks feminine gender on the prominence hierarchy. Importantly, the results observed in all four experiments above revealed that grammatical gender information is organized hierarchically in the same way (masculine over feminine) in both German and French languages. Since previous research on prominence points at the general characteristic of prominence features as cross-linguistically motivated information types that have the same hierarchical organization across languages and linguistic variations (e.g., as it is for animacy in English—McDonald et al., 1993; German—Van Nice and Dietrich, 2003; and Spanish—Prat-Sala, 1997), the results of both studies can be taken as a cross-linguistic validation of gender information as a prominence feature.

In addition to grammatical gender, stereotypical gender information also appears to map onto thematic structure of sentences revealing its own hierarchical structure. Sentences with relative clauses were processed faster when low-ranked patient roles were assigned to stereotypically female nouns and high-ranked agent roles to neutral ones in Experiment 2 in German (Esaulova, 2015). Similarly, stereotypically male agents were relatively easier to process than neutral ones and neutral agents easier than stereotypically female ones in French (Esaulova, 2015). These processing patterns suggest a hierarchy where stereotypically male role nouns outrank neutral ones and neutral role nouns outrank stereotypically female ones thus providing a complementary prominence scale of gender information.

## Gender Prominence: Limitations to be Considered

These findings reveal implicit ways in which grammatical and stereotypical gender affect the interpretation of a thematic structure of a sentence in German and French languages. However, there are several considerations and limitations that need to be taken into account when interpreting the results. Despite differences in the syntactic structure of the experimental materials, grammatical gender appears to be organized in the same hierarchical way and constitute a prominence scale where masculine entities outrank feminine ones. Grammatical gender information affected processing similarly in both German and French languages: feminine entities were perceived as less likely agents/subjects compared to masculine ones. As to the stereotypical gender information, its organization in terms of a prominence scale still remains to be clarified. Due to the properties of the design that allowed the necessary ambiguity in German relative clauses, stereotypically male role nouns were not examined in terms of prominence. Therefore, the position of stereotypically male role nouns is left undefined on the hierarchy in German language, while stereotypically male role nouns outrank neutral ones in French and neutral role nouns seem to outrank stereotypically female role nouns in prominence in both German and French.

Another aspect that needs to be specified regarding gender information as a prominence feature concerns its generalizability. Even though grammatical gender effects appear in sentences with both subject- and object-extracted relative clauses in German, grammatical gender affects the assignment of agent but not patient thematic roles. The design of sentences with backward anaphors in French, on the other hand, allowed for the gender hierarchy regarding the patient but not agent thematic role. Taken together, these effects point at the same hierarchical organization of grammatical gender information in terms of its prominence. However, the direct evidence for grammatical gender to influence the interpretation of agents in French and patients in German sentences is yet to complete the existing results. Moreover, the prominence hierarchy of grammatical gender information observed on role nouns may not be applicable to inanimate or non-human entities. Based on the current evidence, gender hierarchy may be assumed to relate to animacy or even function as its subscale, which brings up further questions about the interaction of gender with other prominence scales.

## Gender Prominence as a Bias

Furthermore, the interdisciplinary nature of the research approach with its potential advantages needs to be considered when evaluating the results of the two studies. Consistent with previous research on gender processing, these studies suggest that both grammatical and stereotypical gender information is used during language processing (e.g., Carreiras et al., 1996; Stahlberg et al., 2007; Reali et al., 2015). At the same time, they go beyond previous research in that they show rather implicit ways in which gender information may influence processing even when it is not explicitly required by the rules of grammatical agreement or in order to resolve the reference. The tendency to associate female/feminine entities with less prominent thematic/syntactic roles and neutral/masculine ones with more prominent roles observed during language comprehension in the two studies can be related to gender hierarchies reported in social psychological research (e.g., Koenig et al., 2011) and may provide an insight on mechanisms underlying gender stereotypes. The social cognitive research on biases has shown that the use of some linguistic structures activates stereotypes, or cognitive expectations and beliefs about a given group of persons. These structures are used preferentially when describing situations that are consistent or inconsistent with a stereotype and thus represent mechanisms that allow stereotypes to be reflected and maintained through language. For instance, Beukeboom et al. (2010) analyzed the use of negations and reported what they call a negation bias—a tendency to use negations when describing behaviors inconsistent with existing stereotypes, such as in saying *not stupid* rather than *smart* when describing a blond girl solving a math problem. Similarly, the use of concrete vs. abstract terms to reflect to which extent behavior was expected or unexpected has been reported as a linguistic intergroup bias (Maass et al., 1989) and an expectancy bias (Wigboldus et al., 2000): adjectives denoting higher levels of abstraction (e.g., *emotional*) tend to be used to encode expected behaviors (e.g., crying women), while unexpected behavior (e.g., crying men) is encoded by action verbs referring to specific events (e.g., *cry*). In the light of this research, the four experiments we described above suggest a bias that reflects and maintains stereotypes about men and women through the thematic structure of a sentence. This bias carries readers’ expectations about gender stereotypes and corresponding social hierarchies (e.g., status, power, agency) over the hierarchy in the thematic structure, so that female/feminine nouns are assigned less prominent (i.e., lower-ranked on a hierarchy and rather passive) patient roles while neutral/masculine nouns are assigned more prominent agent roles. The tendency to perceive nouns possessing certain gender characteristics in one or another thematic role cannot be explained by formal linguistic rules, such as gender agreement, and therefore can be regarded as an implicit gender bias. Previous research has recognized that some information types do bias the assignment of thematic roles (e.g., tendency for animate entities to rather function as agents and inanimate entities as patients) and established them as prominence features. Theoretical argumentation and empirical evidence provided above clearly indicate that gender information can also be conceptualized in terms of prominence hierarchies, even though more extensive research is needed to overcome mentioned limitations and establish gender as a prominence feature.

## Conclusion

This paper proposed theoretical reasoning for grammatical and stereotypical gender of role nouns to be considered a prominence feature and discussed to which extent it is supported by recent empirical evidence from studies in German and French (Esaulova, 2015) languages. Conceptualizing gender as a prominence feature appears beneficial in several ways. First, it theoretically integrates findings on gender effects from different categories of language-based gender information. If gender is considered a supra-linguistic, basic semantic category and a prominence feature, then all linguistic expressions of gender, definitional: *king* vs. *queen*, typical: *soldier* vs. *nurse*, grammatical: *un étudiant* vs. *une étudiante*, should underlie the proposed hierarchical structure and be easier to process when aligned with other prominence features and their respective hierarchical ranks than when unaligned. Secondly, the approach offers an interdisciplinary analysis of gender effects that reflect hierarchical structures in as diverse fields as linguistics and social cognition. Thirdly, the approach allows for new predictions of subtle and implicit gender biases that go far beyond the classic mismatch effects. There is first empirical, cross-linguistic evidence for such biases as reported above.

Taken together, the findings suggest that gender information modulates the accessibility of thematic roles and grammatical functions and thus produces effects similar to those that were previously observed for prominence features, such as animacy. In order to validate the notion of gender as a prominence feature future studies should address questions left open such as the place of stereotypically male entities on a gender hierarchy in relation to stereotypically female and neutral ones, the generalizability of gender hierarchies to other linguistic structures and languages, and the interaction of gender information with other prominence features.

### Conflict of Interest Statement

The authors declare that the research was conducted in the absence of any commercial or financial relationships that could be construed as a potential conflict of interest.

## Footnotes

1 Despite the mapping of grammatical gender to natural gender, which is common for such denotations, they should be regarded as neither perfectly correlated with nor completely independent from one another, as grammatically masculine forms may sometimes be used generically and refer to both men and women.

2 Here and below the term reading times is used to refer to fixations and regressions expressed by a number of eye-tracking measures and on various regions of sentences which are not specified in the text (see the original study for these details).
